# Income-related inequalities in the prevalence of dental pain intensity in adults: gender differences

**DOI:** 10.3389/froh.2025.1510345

**Published:** 2025-01-23

**Authors:** Carolina Veloso Lima, Alanna Barros de Arruda, Mayara dos Santos Noronha, Aline Araujo Sampaio, Marise Fagundes Silveira, Andrea Maria Eleuterio de Barros Lima Martins, Raquel Conceição Ferreira, Aline Netto de Godoy, Daniela Marques da Silva Sousa, Patrick Pereira Garcia, Cyrene Piazera Silva Costa, João Gabriel Silva Souza, Bárbara Emanoele Costa Oliveira

**Affiliations:** ^1^ Department of Restorative Dentistry, Federal University of Paraná, Curitiba, Brazil; ^2^ Graduate Program in Dentistry, University CEUMA, São Luís, Brazil; ^3^ Piracicaba Dental School, University of Campinas, Piracicaba, Brazil; ^4^ Dental School, Federal University of Minas Gerais, Belo Horizonte, Brazil; ^5^ Health Science Programme, University of Montes Claros, Montes Claros, Brazil; ^6^ Dental Research Division, Guarulhos University, Guarulhos, Brazil

**Keywords:** toothache, adult, health inequities, oral health, epidemiological

## Abstract

**Introduction:**

Dental pain is a multifactorial and unpleasant experience that negatively affects daily activities. Previous studies have shown that adults living in socioeconomically disadvantaged areas showed higher dental pain prevalence. This study evaluated whether income inequalities are related to increased dental pain intensity among adults and identified differences between women and men.

**Methods:**

A probabilistic sample of adults was investigated, and income inequality was evaluated using the Gini Index. Dental pain intensity was recorded on a scale from 1–10 for those who experienced dental pain in the 6 months preceding the survey. The covariates were contextual (related to cities) and individual (related to individuals). Associations were investigated for the entire sample and stratified by gender using multilevel Poisson regression models.

**Results:**

Dental pain was reported by 41% of the included sample (*n* = 4,512 adults). Maximum pain intensity was reported more frequently for women than for men. Those living in municipalities with higher Gini Index values reported 1.26 times (95% CI: 1.01–1.56) greater dental pain intensity compared to adults living in cities with lower Gini Index values, even after adjustment by variables. The same pattern was observed when stratified by gender, but it was not statistically significant.

**Conclusion:**

Thus, the contexts of income inequalities can contribute to more severe dental pain intensity among adults. Also, the findings suggest that income inequality does not modify the relationship between gender and dental pain intensity.

## 1 Introduction

Dental pain is a multifactorial, sensory, and emotionally unpleasant experience that negatively affects activities of daily living (1), with high prevalence, mainly in adults (2–6). Considered the most common oral symptom, dental pain affects patients with some oral disease, such as dental caries and periodontal disease (3–8). Interestingly, the presence and intensity of dental pain are not only affected by individual characteristics or by the presence of oral problems but are also modulated by contextual variables (i.e., human development index and gross domestic product) (3, 4, 9–11) and by environmental and political factors (12, 13). Since common oral diseases are unevenly distributed among people living in areas of different socio-economic status (14), the same pattern is expected for dental pain prevalence and its intensity.

Previous studies have shown that adults living in socio-economically disadvantaged areas showed higher dental pain prevalence regardless of their individual characteristics (3, 4, 10, 11, 15). However, the influence of the presence or absence of contextual variables on dental pain intensity has not been well-explored in the adult population. In children, a previous study has found that characteristics of cities, such as the sizes of the municipalities, were associated with dental pain intensity (9), suggesting some relationship between pain intensity and contextual determinants.

Among contextual variables, the Gini Index, a measure of income inequality across a population, has been associated with oral diseases, such as dental caries and periodontal disease (16, 17), use of dental services (18), and oral-health-related quality of life (19), forming a profile of the inequality of distribution. The same behavior should be observed with regard to dental pain, as dental pain is a common consequence of the presence of dental problems and negatively affects the quality of life of those who are more socioeconomically vulnerable (20). Among children, low-income status has been associated with the presence of dental pain in the previous six months (21). For adults, evidence has shown an association between dental pain and low income among young male adults (18 years old) (22), and overall adults (20–59 years old) (20), considering family income. The analysis of economic gradients related to health outcomes can provide important information in terms of the patterns of inequalities, and this may contribute to the design of socially appropriate programs of oral health promotion (12). In this context, the Gini index is an effective measure showing economic gradients across the population and has been used to evaluate global health inequalities (23).

Moreover, the role of gender in the distribution of dental pain is contradictory. Some studies have reported a higher prevalence of dental pain among women than men (3, 24), while others have indicated either the opposite (10, 25, 26) or no difference (27, 28). Therefore, the profile of income inequality in the distribution of dental pain intensity between women and men must be evaluated using a stratified approach, as there is no consensus in the literature regarding the influence of economic gradients on this difference. Therefore, our study (1) evaluated whether dental pain intensity was higher among adults living in cities with increased income inequalities as measured by the Gini Index; and (2) identified if there was a difference in this outcome between women and men by stratified analysis.

## 2 Methods

Data for adults (35–44 years old) from the São Paulo Oral Health 2015 (SBSP-15) survey were used (29). The SBSP-15 was approved by the local Research Ethics Committee (46788215.9.0000.5418), according to the Brazilian National Health Council Resolution for research on human beings. The participants were informed about the study and signed a consent form.

Two-stage sampling clusters with probability proportional to size were adopted. The state of São Paulo, Brazil, was divided into six macroregions (domains). In the first stage, 33 municipalities (primary sampling units) were randomly selected for each domain, except for the metropolitan region of the capital, which included the state capital, and 12 more municipalities were selected (177 municipalities + city of São Paulo). In the second stage, 390 census tracts (second sampling units), 2 sectors for 177 municipalities, and 36 sectors for the capital were randomly selected. All households in each selected census tract were visited to identify individuals in the specified age groups (adolescents, adults, and older people). The exhaustion technique (until the estimated sample was achieved) was used for each primary sampling unit. The sample size was defined to estimate the prevalence of the main dental conditions. The parameters for sample size calculation were DMFT mean, prevalence of periodontal disease, or prevalence of dental prosthesis use, according to the Brazilian National Survey for the Southeast Region. Also considered were: *ε* = 0.10, deff = 2, and non-response rate = 30%.

The data were collected by interview and oral examination according to World Health Organization (30) and the Brazilian national survey—SB Brazil 2010 (31) guidelines in terms of indexes, ages, and sampling. Dentists previously trained and calibrated (Kappa value = 0.65 for each examiner) by the consensus technique conducted the oral examinations. In the present study, only adults (35–44 years old) who answered the question about dental pain intensity were included. All analyses were performed for the total sample and stratified by gender (women and men).

### 2.1 Dependent variable

The dependent variable “dental pain intensity” was evaluated by two sequential questions. First, the individuals were asked, “Have you had dental pain in the past 6 months?” (Yes, No, and I do not know/Did not answer). Participants who answered “Yes” quantified the pain intensity on a scale from 1–10, where was explained to each individual that “1” meant “Very little pain” and “10” meant “Very strong pain”. Individuals who answered “no dental pain” in the previous 6 months (first question) were considered “0” on the intensity scale. The answer “I do not know” or those who did not answer were considered as missing values. Dental pain intensity was analyzed as a numerical variable.

### 2.2 Exploratory variable and covariates

Income inequality, the main exploratory variable, was evaluated by the 2010 Gini Index at the municipal level. This Index has been used to measure health inequalities related to income across a population. The Gini Index was extracted from the *Atlas Brasil* Web site for each studied municipality. The coefficients ranged between 0 (total equality) and 1 (total inequality) (32). In this study, the Gini Index was dichotomized according to the total average of municipalities evaluated in “up to average” and “higher than average”.

From public online databases, the following contextual variables were considered: Human Development Index (HDI); percentage of households with access to piped water; percentage of oral health coverage teams available in the primary care of the public service; number of dentists per 1,000 inhabitants; presence of Dental Specialized Centers in the city; and presence of fluoridated water in the city. The HDI scores of municipalities in 2010 were retrieved from the *Atlas Brasil* website. We collected information about oral care from the General Coordination of Oral Health (*Coordenação Geral de Saúde Bucal—Brasil Sorridente*) database. The information about the presence of fluoridated water in the city (in the period from 2014–2015) was collected from the Collaborating Center of the Ministry of Health in Oral Health Surveillance (CECOL) report. All contextual variables were considered as numerical variables (quantitative).

**Figure 1:**
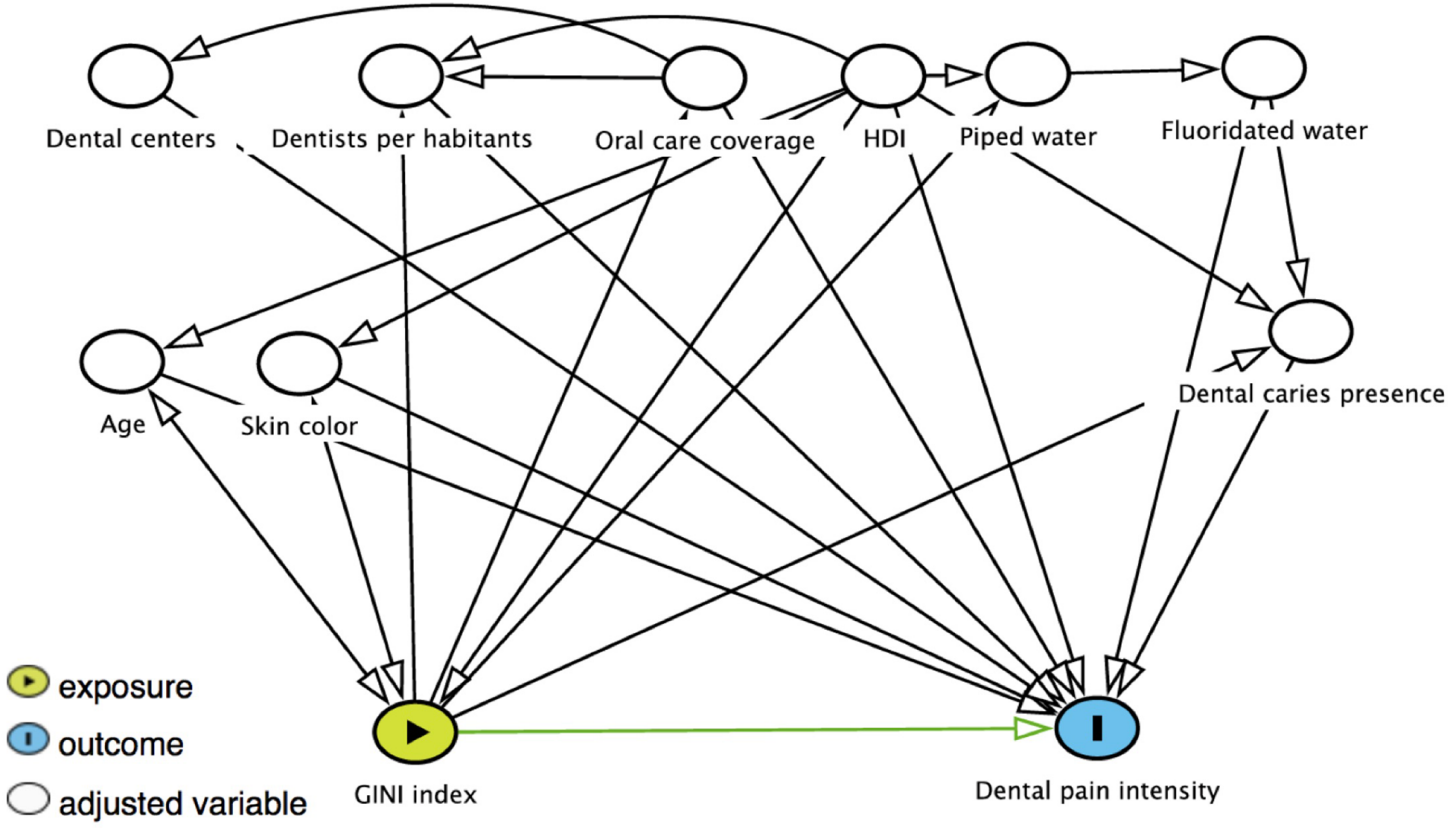
Directed Acyclic Graph (DAG) for associations between the Gini Index, contextual and individual variables, and dental caries presence and their relation with dental pain intensity in adults. HDI, human development index.

These contextual variables were chosen because they may be related to the Gini Index and dental pain, as illustrated in the Directed Acyclic Graph (Figure 1) that shows the directions of possible relationships between variables. The Human Development Index (HDI) measures a country's progress in three fundamental dimensions of human development: income, education, and health. The availability of piped water is positively associated with oral health (33). Limited access to dental services is linked to poorer oral health outcomes among socially disadvantaged adults, while equitable access can mitigate the effects of socioeconomic disparities on health (34). In Brazil, public oral health services are provided free of charge to the population by the health system, primarily in disadvantaged areas. Primary oral care includes promotion and prevention strategies, as well as clinical care, provided free of charge by Oral Health Teams consisting of a dentist and an oral health assistant or technician. Each team is responsible for a specific number of people within a given area. Dental Specialized Centers is also part of public health system in the country. The other variable measures the number of dentists per inhabitant in a given location, both in the public and private sectors. It is hoped that a larger number of dentists will be able to meet the needs of the population more comprehensively (35). Finally, the presence of fluoridated water is associated with improved oral health and reduces the impact of socioeconomic inequalities on oral health (36).

The individual variables considered were the number of teeth with dental caries, age group (35–49 and 40–44 years), and skin color (white or non-white). The incidence of dental caries was extracted from the Decayed-Missing-Filled Teeth index (DMFT) evaluated according to WHO.

### 2.3 Statistical analysis

A directed acyclic graph (DAG) was constructed from the theoretical framework for dental pain to guide the selection of covariates for adjusting the associations of interest (37, 38) (Figure 1). The DAG is an important tool for reducing estimate bias through the selection of covariates and model adjustment involving exposure (Gini Index) and an outcome (dental pain). In this model, the contextual variables (environmental characteristics) were directly or indirectly related to dental pain intensity. Moreover, individual variables, represented by demographic characteristics, were directly related to the outcome and could be affected by contextual variables. The presence of dental caries has been considered the main reason for the presence of dental pain, and this variable is directly associated with dental pain intensity. However, the presence of dental caries is also modulated by contextual and individual variables. Therefore, the DAG shows a main outcome (dental pain) and main exposure factor (GINI index). The independent variables related to the outcome and exposure are selected based on previous evidence (37, 38). The direction of the arrows indicates the factors that affect the other variable.

The data were analyzed by STATA version 15.1. Descriptive analyses were performed to estimate frequencies of the investigated variables. Multilevel Poisson regression (fixed-effect and random-intercept) models were used. A sequence of multilevel models was adjusted by the DAG model (Figure 1). The first (“empty”) model included only the dependent variables (model 1). A significant random-intercept variance indicated the presence of unexplained differences in dental pain intensity between municipalities. The Wald test evaluated the significance of random intercepts, and the Median Rate Ratio (MRR) measured heterogeneity among municipalities (39). There was no variation among municipalities when the MRR was 1.0, but the higher the MRR, the greater the area-level variation. The second model (model 2) included only the Gini Index as exposure. The third model (model 3) included the Gini Index variable and other contextual variables. Model 4 included the Gini Index and contextual and individual determinants. The final model (model 5) considered all independent variables: the Gini Index, contextual and individual variables, and the presence of dental caries. The MRR was used to assess the reduction of variations among municipalities as the variables were included in the model (39). An MRR equal to 1.0 indicated no heterogeneity between the contexts analyzed. All analyses were performed based on correction for the design effect and sample weight for each individual.

## 3 Results

**Figure 2:**
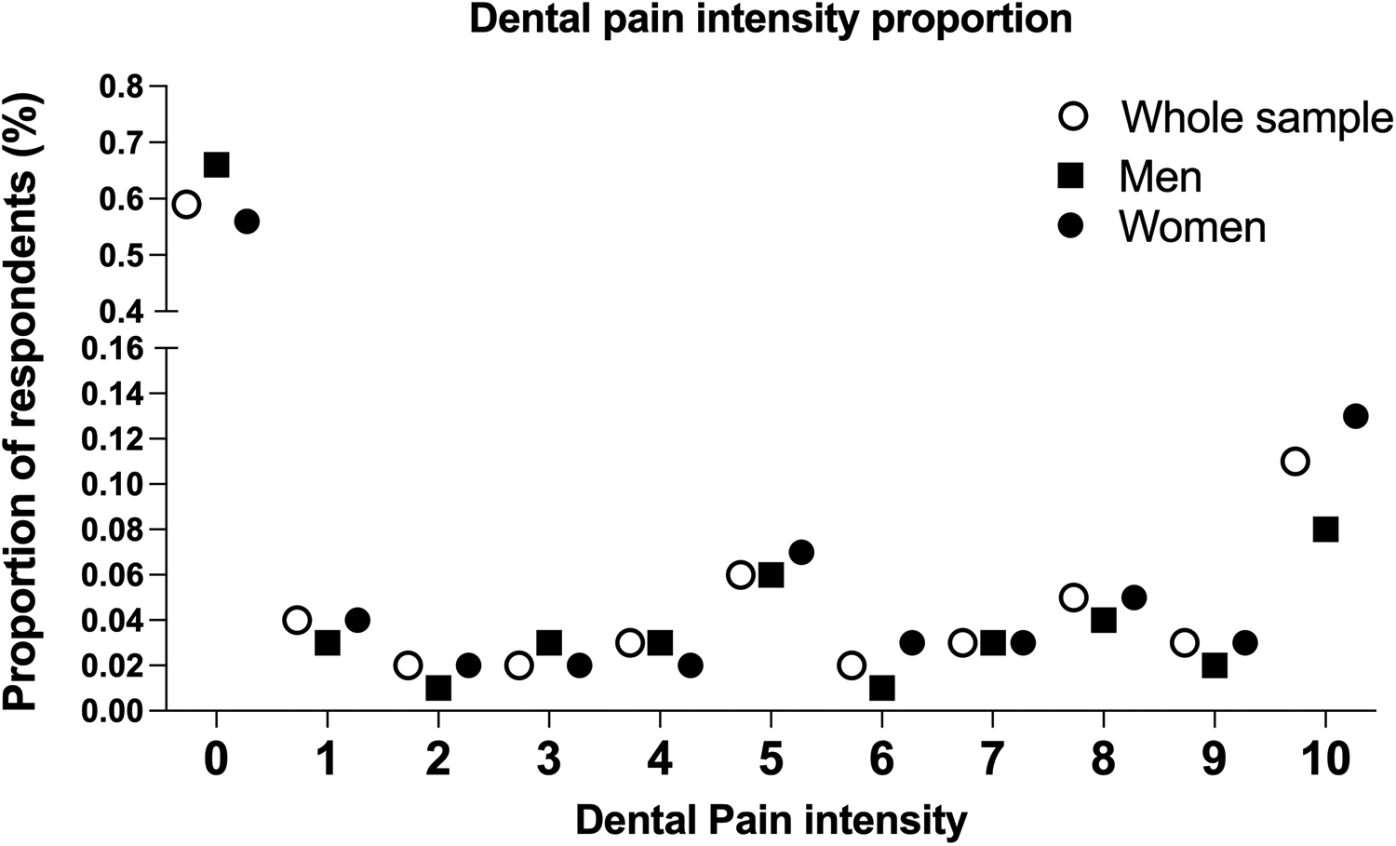
Proportion of dental pain according to intensity in adults, based on the entire sample of women and men.

In total, 4,512 adults answered the question about dental pain, representing 74.5% of the total sample (*n* = 6,051). Women equaled 70% of the total sample. Forty-one percent (34%, men; 44%, women) reported having experienced some degree of dental pain in the six months preceding the survey (Figure 2). Maximum pain intensity (scale = 10) was reported more frequently for women (12%) than for men (7%) (Figure 2). Overall, adults were living in cities with good contextual parameters, with a Gini Index of up to 0.42 (Table 1).

**Table 1:** Descriptive analysis of contextual and individual variables in adults of the São Paulo state (Brazil) relative to the total sample and by gender (women and men) and crude prevalence ratio according to dental pain intensity. *N* = 4,512 (total sample), 3,097 (women) and 1,415 (men).

	Total sample		Women		Men	
	Proportion or average	Crude PR (95% CI) Dental pain intensity	Proportion or average	Crude PR (95% CI) Dental pain intensity	Proportion or average	Crude PR (95% CI) Dental pain intensity
Gini Index						
Up to 0.42	0.71	1	0.72	1	0.70	1
Higher than 0.42	0.29	1.26 (1.01–1.56)	0.28	1.23 (0.98–1.53	0.30	1.30 (0.94–1.80)
CONTEXTUAL						
HDI	0.76	0.28 (0.007–9.97)	0.76	0.26 (0.007–9.64)	0.76	0.10 (0.0005–21.50)
Provided with piped water^a^	97.7	0.99 (0.96–1.02)	97.4	0.99 (0.96–1.02)	97.6	0.99 (0.95–1.04)
Coverage for oral health in primary care^a^	42.2	1.00 (0.99–1.00)	41.0	1.00 (0.99–1.00)	43.4	1.00 (0.99–1.01)
Dentists per 1,000 inhabitants^a^	0.80	0.95 (0.76–1.20)	0.77	0.99 (0.79–1.25)	0.85	0.87 (0.63–1.22)
Fluoridated water						
No	0.01	1	0.01	1	0.01	1
Yes	0.99	0.75 (0.37–1.51)	0.99	0.75 (0.37–1.51)	0.99	0.61 (0.22–1.69)
Access to Dental Specialized Centers						
No	0.32	1	0.33	1	0.30	1
Yes	0.68	0.99 (0.93–1.06)	0.67	0.99 (0.92–1.07)	0.70	0.97 (0.85–1.11)
INDIVIDUAL						
Age (years)						
35–39	0.53	1	0.53	1	0.52	1
40–44	0.47	0.99 (0.98–1.00)	0.47	0.99 (0.98–0.99)	0.48	1.01 (1.00–1.02)
Skin color						
White	0.60	1	0.58	1	0.64	1
Non-white	0.40	1.19 (1.15–1.24)	0.42	1.22 (1.17–1.28)	0.37	1.12 (1.03–1.22)
ORAL HEALTH						
Number of teeth with dental caries^a^	1.67	1.06 (1.06–1.07)	1.61	1.06 (1.06–1.07)	1.83	1.08 (1.07–1.09)

HDI, human development index. Proportion for categorical variables and average for numerical variables. CI, confidence intervals; PR, prevalence ratio.

^a^ Variables described by average.

Those living in municipalities with a Gini Index higher than 0.42, that is, greater income inequality, reported more severe pain than did those living in municipalities with a Gini Index up to 0.42 [prevalence ratio (PR), 1.26; 95% CI, 1.01–1.56]. Similar results were also found for both genders in terms of PR (Table 1). Interestingly, compared with the empty model (model 1), the MRR and the variance fell from 0.46–0.44 (PCV = −4.34%) after the inclusion of the Gini Index, indicating that this contextual variable contributed little to explanation of the variations between municipalities in the distribution of dental pain severity. The adjustment of the model for other contextual variables did not change this relationship and contributed little to explanation of the distribution of pain intensity among the municipalities.

The association between the Gini Index and dental pain intensity was statistically significant (*p* < 0.05) independently of the individual variables in the total sample (Table 2). The same was not observed for men and women. After the inclusion of the dental caries variable (model 5), no significant association (*p* < 0.05) was observed between the Gini Index and dental pain intensity. Overall, all models for the entire sample showed higher dental pain intensity for adults living in areas with higher income inequality as measured by the Gini Index (Table 2). Although not statistically significant (*p* > 0.05), the same pattern was found for stratified analysis for women and men, showing higher dental pain intensity for women and men living in cities with a higher Gini Index (Table 2).

**Table 2:** Prevalence ratio (95% confidence intervals in parentheses) of dental pain intensity in multilevel models with random intercepts and fixed effects according to the gini Index (higher than 0.42—average) and adjusted by contextual and individual determinants and dental caries presence in 35- to 44-year-olds. *N* = 4,512 (total sample), 3,097 (women) and 1,415 (men).

	Model 1		Model 2		Model 3		Model 4		Model 5	
	PR (95% CI)	*P*	PR (95% CI)	*P*	PR (95% CI)	*P*	PR (95% CI)	*P*	PR (95% CI)	*P*
Entire sample										
Gini Index	–	–	1.26 (1.01–1.56)	.**038**	1.27 (1.02–1.59)	.**033**	1.25 (1.00–1.56)	.**044**	1.23 (0.99–1.53)	.060
Variance	0.46		0.44		0.44		0.43		0.42	
MRR	1.91		1.89		1.88		1.88		1.86	
Women										
Gini Index	–	–	1.23 (0.98–1.53)	.064	1.25 (1.00–1.56)	.**048**	1.23 (0.98–1.54)	.065	1.20 (0.96–1.50)	.095
Variance	0.45		0.44		0.44		0.44		0.43	
MRR	1.90		1.89		1.88		1.88		1.87	
Men										
Gini Index	–	–	1.30 (0.94–1.80)	.109	1.30 (0.93–1.80)	.113	1.28 (0.92–1.78)	.128	1.27 (0.92–1.76)	.141
Variance	0.90		0.88		0.84		0.84		0.83	
MRR	2.47		2.45		2.40		2.40		2.39	

Model 1—empty.

Model 2—only the Gini Index variable.

Model 3—Gini Index + contextual variables.

Model 4—Gini Index + contextual variables + individual variables.

Model 5—Gini Index + contextual variables + individual variables + dental caries.

Contextual variables: HDI, provided with piped water; Coverage for oral health in primary care; Dentists per 1,000 inhabitants; Access to fluoridated water; Access to Dental Specialized Centers. Individual variables: age and skin color.CI, confidence intervals; PR, prevalence ratio; MRR, median rate ratio.

## 4 Discussion

Our findings showed that Brazilian adults living in disadvantaged areas in terms of income inequality, as measured by the Gini Index, had more severe dental pain than adults living in conditions of greater income equality, even after adjustment by contextual and individual variables. To the best of our knowledge, this is the first study that has assessed the relationship between dental pain intensity and income inequality among adults, as measured by the Gini Index, adjusted by other exploratory variables. Although previous studies have evaluated the association between dental pain and contextual aspects (3, 4, 10, 11, 40), pain has been evaluated simply as either present or absent. However, the evaluation based on pain intensity can show properly the severity of this outcome and highlight important inequalities in its distribution.

Regarding contextual variables, it is known the poorest and most marginalized in society are disproportionately affected by oral diseases (37) including dental pain, which is unequally distributed across social and economic strata (20). This relationship between income and dental pain is also evident throughout life, with early exposure to relative poverty leading to a greater experience of pain in adulthood (41). Also, characteristic “cities” has also been associated with dental pain in children, with lower intensity reported for children living in smaller than in bigger cities (9). Considering that lower income distribution inequality is expected in small cities (9), our results for adults followed a similar pattern. Some implications should be considered, since individuals living in disadvantaged areas might encounter barriers when seeking treatment, as well as reduced access to dental services due to cost. This is supported by Bhandari et al. (2014) (42) who reported that the Gini index is negatively correlated with use to dental services. Therefore, the impact of social gradients on health outcomes may provide valuable insights for policymakers. Implementing public policies to manage pain and its symptoms in the adult population is essential. Preventive strategies targeting major oral diseases that cause pain, along with therapeutic and rehabilitative interventions for individuals already affected, can help address the link between dental pain and socioeconomic inequalities. Increasing and facilitating access to health services, particularly for those in the lowest socioeconomic strata, can play a critical role in reducing social inequalities. Therefore, it is important to develop or improve oral health policies that simultaneously provide the following: (1) preventive strategies based on oral health literacy to develop health behaviors that control or prevent oral problems leading to dental pain; (2) increased access to dental services for therapeutic and preventive reasons, particularly in poorer areas and among disadvantaged groups previously identified and addressing barriers to dental service access; (3) monitoring of factors affecting oral health outcomes to enhance current strategies and collect epidemiological data from the population to estimate the need for dental services and reorganize services and strategies; (4) development of ways to provide essential tools for health control, such as access to fluoride toothpaste and tooth brushing for people living in disadvantaged areas.

In Brazil, the public health system, focusing on disease prevention and health promotion, has among its main purposes the reduction of health inequalities, including those related to oral health (43). However, in terms of oral health care, although important advances have been achieved since the implementation of the public health system, some issues related to universal access to services remain to be improved (44, 45). Strategies such as reducing waiting times at health centers, offering care at flexible hours, expanding the oral health care network, and training professionals are among the interventions that can help reduce dental pain in underserved populations.In fact, in our model, even after adjustment by other contextual variables related to public dental services (Dental Specialized Centers and coverage of oral health in primary care), the relationship between the Gini Index and dental pain intensity was maintained. As an obstacle to be overcome by the public health system, the reduction of income-related inequalities needs to be addressed by reducing the burden of oral diseases and their impact, such as dental pain intensity.

Dental pain has also been previously associated with dental caries (46, 47), and people with this condition have been reported to have 56 times more chance of having pain (3). This strong relationship between dental caries and dental pain may mask the association with other individual and contextual variables that explain in the higher heterogeneity of the model after inclusion of the dental caries variable. The adults included in this study had an average of 1.6 teeth with untreated dental caries, which probably led to dental pain, mainly for lesions with proximity to the dental pulp. The higher heterogeneity of the model after inclusion of the dental caries variable may explain the lack of a relationship between the Gini Index and dental pain intensity found in model 5. However, the same pattern was found for the prevalence ratio, showing higher dental pain intensity in adults living in cities with a higher Gini Index.

Moreover, although prevalence showed the same pattern of results for both genders, women reported more intense dental pain than did men. The slight difference in the 95% CI between women and men may be explained by the higher range of pain intensity for men. It is important to note that there were more women in the survey, which could explain the higher dental pain prevalence among women. Despite that, the prevalence of dental pain associated with gender should be carefully considered, since it remains controversial in the literature and may be affected by sample characteristics (3, 10, 21). This difference can also be justified by social (48), biological, and behavioral factors (28), with cultural and environmental factors might predispose women to more frequent pain symptoms (48). Women are more susceptible to chronic pain and have greater sensitivity to pain. Several biopsychosocial mechanisms may be involved in these gender differences in toothache, including sex hormones, endogenous opioid function, genetic factors, coping mechanisms, and gender roles (49). Some studies reporting that women are more sensitive to body discomfort and seek more dental care compared with men (22, 50, 51). In addition, the perception of chronic pain is higher among women, especially those from lower socio-economic backgrounds (51). Regarding dental pain, women with lower socioeconomic status experience a greater impact this condition that affects their daily activities (20). Gender differences in the relationship between income inequality and health have been reported in the literature and are often directly linked to cultural context (52). In our study, women and men showed that dental pain intensity was higher among adults living in disadvantaged areas, regardless of gender. Although some gender differences have been reported for oral problems and symptom outcomes (3, 10), our results suggest that living in an area characterized by inequality may reduce gender differences for dental pain intensity, and all individuals are affected by inequalities. Moreover, healthcare programs targeting specific groups, such as women and individuals at certain ages, may also help reduce health-related inequalities.

This study presented some limitations, since the database from a cross-sectional design was used and did not allow us to make a temporal association between dental pain intensity and contextual and individual factors, thereby also not allowing us to identify a cause-effect relationship. Moreover, the sample was stratified, which may have reduced its power for revealing associations among variables. In fact, there were fewer men than women in the sample, reducing the numbers in each city in the multilevel model, which may have affected the range of some parameters, such as 95% CI. Additionally, these parameters could also have been affected by the high number of cities in the multilevel model. Thus, an extrapolation of these findings to other populations must be undertaken with caution. Importantly, MRR was used to evaluate the variations among municipalities, as the variables were included in the model in terms of heterogeneity. The values found suggest small changes across the models and a slight reduction in the variations when comparing Model 1 and Model 5. Furthermore, the use of secondary data impairs the evaluation of other explanatory variables relevant to the outcome. Further studies also need to explore these outcomes with larger samples, particularly for men. Importantly, the presence and intensity of dental pain may reflect limited access to dental services for pain management. In this study, we considered contextual variables to measure access to oral care services, such as the percentage of oral health coverage teams available in the primary care of the public service and the presence of Dental Specialized Centers in the city. However, at the individual level, the characteristics of dental service access—such as the reasons for seeking care, the type of service accessed, and their relationship with pain intensity—require further exploration. Furthermore, pain is a multidimensional construct influenced by physical, social, and psychological factors. It can also be modulated by previous experiences, location, and intensity. Therefore, the relationships established in this study for individuals living in disadvantaged areas should be explored in greater detail to develop effective preventive and control measures.

Future research should be conducted with longitudinal designs, primarily to establish whether there is a cause-and-effect relationship between income inequalities and the individual and contextual factors associated with dental pain perception, particularly in comparison to people living in advantaged areas. Additionally, it should explore why women might report more severe dental pain than men, providing new insights into specialized oral health care measures directed by gender. Moreover, other measures of inequality and social gradients must be considered in the evaluation of dental pain intensity among adults.

## 5 Conclusion

In conclusion, our findings showed that adults living in cities with higher income inequalities, as measured by the Gini Index, showed a higher prevalence of more intense dental pain than those who lived in cities with lower income inequalities, even after adjustment by other exploratory contextual and individual variables. The same pattern was observed in stratification by gender. Further research should explore longitudinal designs to evaluate the cause-and-effect relationship based on the findings reported here and include more details regarding pain evaluation and income inequalities to unravel this association.

## Data Availability

The original contributions presented in the study are included in the article/Supplementary Material, further inquiries can be directed to the corresponding authors.
